# *In Vitro* Seamless Stack Enzymatic Assembly of DNA Molecules Based on a Strategy Involving Splicing of Restriction Sites

**DOI:** 10.1038/s41598-017-14496-5

**Published:** 2017-10-27

**Authors:** Dong Yu, Yanning Tan, Zhizhong Sun, Xuewu Sun, Xiabing Sheng, Tianshun Zhou, Ling Liu, Yi Mo, Beibei Jiang, Ning Ouyang, Xiaolin Yin, Meijuan Duan, Dingyang Yuan

**Affiliations:** 1 State Key Laboratory of Hybrid Rice, Hunan Hybrid Rice Research Center, 736 Yuanda Rd, 410125 Changsha, China; 2 Hunan Academy of Agricultural Sciences, 892 Yuanda Rd, 410125 Changsha, China; 3 College of Bioscience and Biotechnology, Hunan Agricultural University, 1 Nongda Rd, 410128 Changsha, China; 4 Long Ping Branch, Graduate School of Hunan University, 892 Yuanda Rd, 410125 Changsha, China

## Abstract

The standard binary enzymatic assembly, which operates by inserting one DNA fragment into a plasmid, has a higher assembly success rate than the polynary enzymatic assembly, which inserts two or more fragments into the plasmid. However, it often leaves a nucleotide scar at the junction site. When a large DNA molecule is assembled stepwise into a backbone plasmid in a random piecewise manner, the scars will damage the structure of the original DNA sequence in the final assembled plasmids. Here, we propose an *in vitro* Seamless Stack Enzymatic Assembly (SSEA) method, a novel binary enzymatic assembly method involving a seamless strategy of splicing restriction sites via a stepwise process of multiple enzymatic reactions that does not leave nucleotide scars at the junction sites. We have demonstrated the success and versatility of this method through the assembly of 1) a 4.98 kb DNA molecule in the 5′ → 3′ direction using BamHI to generate the sticky end of the assembly entrance, 2) a 7.09 kb DNA molecule in the 3′ → 5′ direction using SmaI to generate the blunt end of the assembly entrance, and 3) an 11.88 kb DNA molecule by changing the assembly entrance.

## Introduction

Synthesis of medium-sized and even large DNA molecules is increasingly necessary for gene function research^1,2^. An essential requirement in synthetic biology is the assembly of multiple, small DNA fragments into large constructs in a defined order and orientation^3^. Traditional methods rely on restriction enzyme digestion followed by ligation, an approach that works well for the insertion of single, short DNA sequences into vectors; however, it is challenging to identify enough distinct restriction sites to clone long DNA fragments and piecewise ligation often leaves scars at the junction sites^4^. Several applications using type IIS restriction enzymes have been developed to seamlessly join DNA sequences, such as Golden Gate cloning^5,6^ and Pairwise Selection Assembly (PSA)^7^, but are still limited by type IIS restriction sites present in the assembled sequence^8,9^. Site-specific recombination has been widely used to clone a broad range of genes and elements, in part, because these methods do not rely on restriction sites. However, the limitation of site-specific recombination is the requirement for construction of multiple entry vectors and the inability to achieve seamless ligation^4,10,11^. A seamless strategy of ligation-independent cloning (LIC) has also been developed^12,13^. LIC technologies are dependent on overlapping homologous sequences at the ends of DNA fragments^4^, including polymerase cycling assembly (PCA)^14^, circular polymerase extension cloning (CPEC)^15^, advanced quick assembly (AQUA) cloning^16^, and enzymatic reaction assembly^17–19^. Enzymatic reaction assembly has become increasingly popular for gene synthesis and vector construction due to its ease of use and its flexibility in one-step cloning of multiple fragments. Its popularity has been increased by the development of reliable commercial kits, such as the In-Fusion® HD Cloning Kit, Gibson Assembly® Cloning Kit, and NEBuilder HiFi DNA Assembly kit, and pEASY-Uni Seamless Cloning and Assembly Kit. However, the polynary enzymatic assembly method, which inserts two or more fragments into the plasmid, may not be successful in some applications because it has a variable success rate that depends on the number of inserted fragments^19^ and may produce mutants in the final products due to PCR errors^20^. The standard binary enzymatic assembly method has a higher success rate than the polynary method, but it is challenging to assemble a DNA molecule larger than 3.5 kb in a plasmid due to the limitation of long PCR amplification for complex genomic DNA or cDNA templates. When a large DNA molecule is assembled in a random piecewise manner using the standard binary enzymatic assembly method, it often leaves a nucleotide scar at the junction between two adjacent pieces^21^. These scar sequences will damage the structure of the overall DNA sequence^22^. To overcome these shortcomings, we propose a Seamless Stack Enzymatic Assembly (SSEA), an alternative, seamless enzymatic DNA assembly method involving splicing of restriction sites that allows for DNA fragments to be inserted into a vector stepwise over multiple rounds of enzymatic reactions. SSEA is different from previous enzymatic assembly methods—such as polynary enzymatic assembly employing In-Fusion^®^ HD Cloning and Gibson Assembly^®^ Cloning kits—that join more than 3 DNA fragments together to form a final large DNA molecule in one reaction. The core principle of SSEA is to utilize stitching sites, which are portions of the restriction site nucleotides existing in the assembled sequence, to segment the large DNA molecule into specific regions. These stitching sites are included when designing primers, and the stitching sites help restore the pre-existing restriction site in the assembly products by splicing to the end of the linearized plasmid. The restored restriction site can be cut to generate the assembly entrance for the next round of enzymatic reactions so that there is not a scar at the junction site between two adjacent fragments. As an experimental demonstration, a 4.98 kb DNA sequence that was reverse complemented with Chr.3: 3700958-3705941 in *Oryza Sativa Japonica* L. was seamlessly assembled stepwise into an expression vector in 3 rounds of enzymatic reactions. We have also succeeded in assembling a 7.09 kb DNA sequence and an 11.88 kb DNA sequence in 2 and 5 rounds of reactions, respectively.

## Results

### The protocol for SSEA

**Figure 1:**
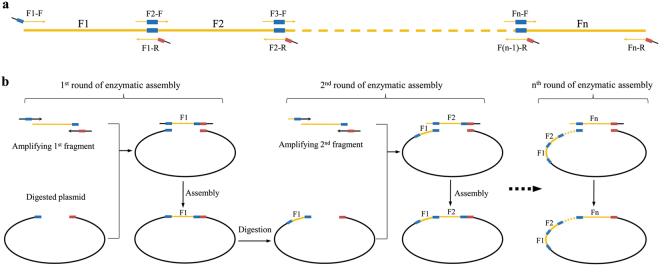
Schematic diagram illustrating the design of SSEA. The black line represents pLDR and orange represents the assembled DNA sequence. The thick short line shown in blue and red represents the 2 stitching sites which could be spliced together to regenerate a restriction site. (**a**) The assembled DNA sequence was divided into several fragments by stitching sites and the orange arrows represent the matching primers with overlapping. (**b**) After each round of PCR amplification, digestion, and assembly, the original restriction site is regenerated only once in the construct, which then serves as the assembly entrance for the next fragment. By iteration of these steps, a long DNA sequence can be cloned into a plasmid with high fidelity, but without introducing scar sequences.

The protocol for SSEA is as follows: (i) An appropriate restriction enzyme is selected to be used as the assembly entrance enzyme; (ii) The selected restriction enzymes were used to linearize the plasmid, providing an entrance for assembly of the DNA fragment. The linearized plasmids include portions of the restriction site on both ends, and the portions of restriction sites are termed stitching sites. The corresponding stitching sites of several common restriction enzymes present in the backbone vector of pLDR are listed in Table 1 (for details of pLDR see Supplementary Figure S1); (iii) The DNA assembly direction is determined according to the appropriate stitching site and several stitching sites are selected as the boundaries dividing the larger DNA molecule into several shorter DNA fragments; (iv) A set of overlapping primers are designed for amplifying the fragments; (v) The plasmid is linearized with the assembly entrance enzyme and the assembly fragments are amplified via PCR; (vi) The first fragment is enzymatically assembled in the linearized plasmid that restores an assembly entrance used for the second round of seamless assembly in the newly assembled plasmid; and (vi) Steps (v) and (vi) are repeated to assemble all of the fragments in the plasmid. Figure 1 illustrates the schematic of SSEA.

**Table 1:** Stitching site of several common restriction enzymes present in pLDR.

Restriction enzymes	Recognition sequence	Stitching sites corresponding to assembly direction	
		5′ → 3′	3′ → 5′
HindIII	AAGCTT	AAGCT	AGCTT
PstI	CTGCAG	CTGCA	TGCAG
SalI	GTCGAC	GTCGA	TCGAC
XbaI	TCTAGA	TCTAG	CTAGA
BamHI	GGATCC	GGATC	GATCC
SmaI	CCCGGG	CCC	GGG
KpnI	GGTACC	GGTAC	GTACC
SacI	GAGCTC	GAGCT	AGCTC
EcoRI	GAATTC	GAATT	AATTC

### Case 1: Assembly of a 4.98 kb DNA molecule by generating the sticky end of the assembly entrance

**Figure 2:**
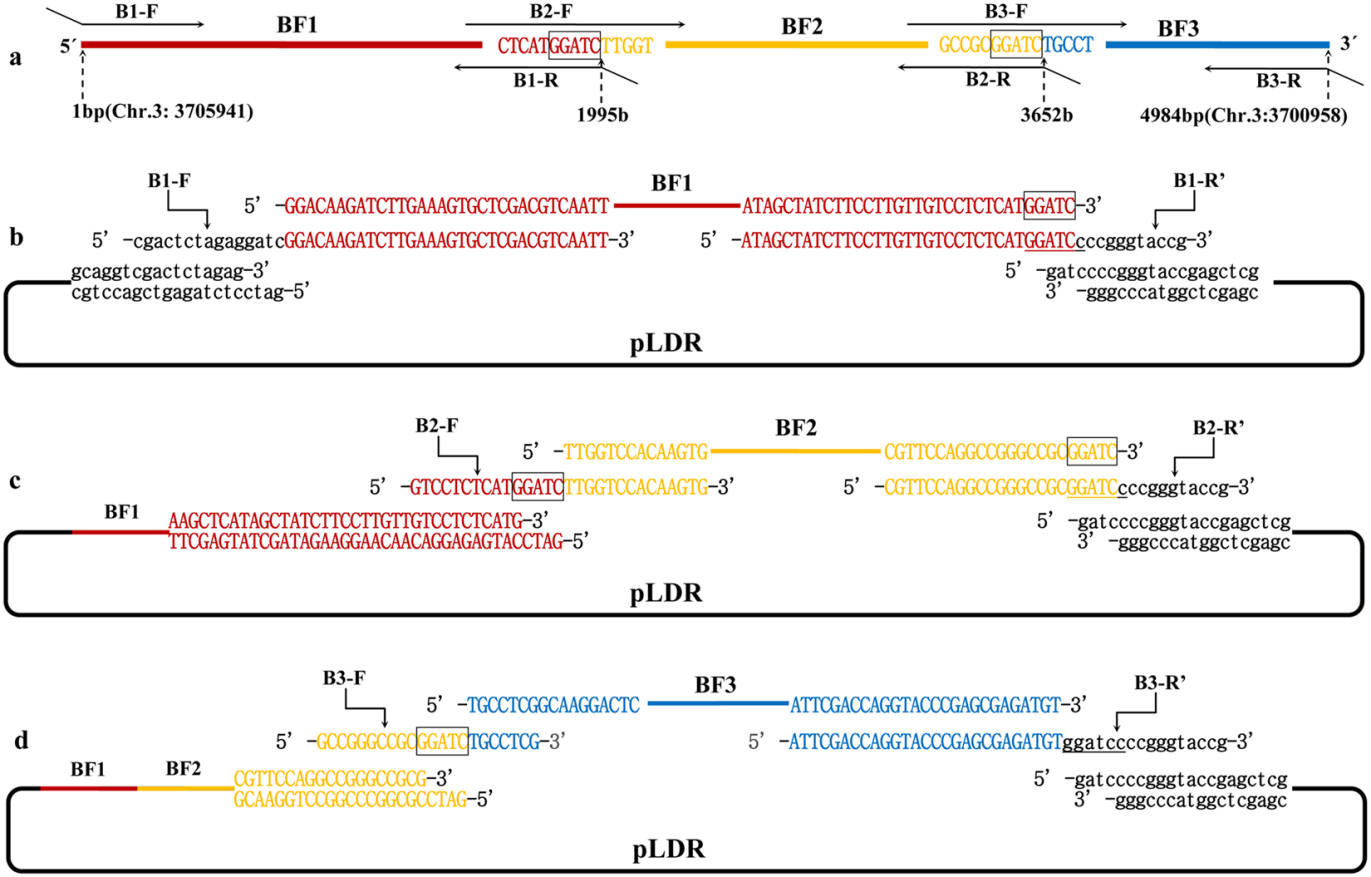
Assembly of a 4.98 kb DNA molecule by generating the sticky end of the assembly entrance. The stitching sites GGATC are boxed. The line and lowercase sequence shown in black represents pLDR. The line and uppercase sequence shown in red represents the first fragment of BF1, orange represents the second fragment of BF2, and blue represents the third fragment of BF3. (**a**) The 4.98 kb DNA molecule was divided into 3 fragments by 2 stitching sites GGATC and the matching overlapping primers. (**b**) The first round of the enzymatic assembly reactions for inserting BF1 is shown. The backbone vector of pLDR was digested by BamHI. The BamHI site was then eliminated in B1-F, and a BamHI site (GGATCc) was restored by splicing the first stitching site and overlapping region in B1-R′ (B1-R′ was a reverse complement with B1-R). When the BF1 amplified with primers of B1-F and B1-R was assembled into pLDR, there was only 1 BamHI site in the assembled products, which could be cut to generate the assembly entrance for the second round. (**c**) The second round of the enzymatic assembly reaction for inserting BF2 is shown. The intermediate plasmid of pLDR-BF1(4) was digested by BamHI. There was a BamHI site (GGATCc) spliced by the second stitching site and overlapping region in B2-R′ (B2-R′ was a reverse complement with B2-R), but there was no BamHI site spliced in B2-F despite the first stitching site GGATC existing in B2-F. As for the first stitching site GGATC in B2-F, the BF2 must join together with the BF1 seamlessly, and then there is only 1 BamHI site in the assembled products, which could be cut to generate the assembly entrance for the third round. (**d**) The third round of the enzymatic assembly reaction for inserting BF3 is shown. The intermediate plasmid of pLDR-BF1 ~ BF2(1) was digested by BamHI. As for the second stitching site GGATC in B3-F, the BF3 must join together with the BF2 seamlessly. A BamHI site was retained in the overlapping region of B3-R′ for other consequent applications (B3-R′ was a reverse complement with B3-R).

A 4.98 kb sequence reverse complemented with Chr.3: 3700958-3705941 in *Oryza Sativa Japonica* L. is the genome DNA of the *CYP704B2* gene including its promoter, CDS, and its terminal. There are 44 restriction sites, including the common restriction sites of BamHI, PstI, SpeI, and XbaI present at the multiple cloning sites of pLDR, that were absent in the 4.98 kb DNA sequence analyzed by DNAssit 2.0 (Supplementary Figure S2). We selected BamHI to linearize the plasmid to generate the assembly entrance and determine the 5′ → 3′ assembly direction according to the stitching site GGATC. There were 6 stitching sites GGATC present in the 4.98 kb DNA molecule. Two stitching sites GGATC were identified and used to divide the 4.98 kb DNA molecule into 3 pieces of DNA fragments, with the size of the 3 fragments being 1995 bp, 1657 bp, and 1332 bp (Fig. 2a). Three pairs of primers were designed to amplify the 3 pieces of DNA fragments (Table 2). The forward primer of the first fragment was designed to eliminate the BamHI site, and the reverse was designed for splicing a BamHI site by stitching site and overlapping region to restore a restriction site in the first assembled product. The forward primer of the second fragment was at or near the junction of the first stitching site, 1 side of the junction was 15 bp overlapped with the first fragment and the other side was a special sequence of the second fragment. The second reverse primer was designed similarly to the first reverse primer while the third pair of primers was designed similarly to the pair for the second fragment.

**Table 2:** The primer sequence.The underlined sequence shown in 1 and 1’, 2 and 2’, 3 and 3’, 4 and 4’, 5 and 5’, 6 and 6’, 7 and 7’ are reverse complements. The boldface sequence gatcc, which was added to reserve a BamHI site in the overlapping region in B3-R, did not destroy the structure of the assembled sequence of the 4.98 kb DNA molecule in the final plasmid. The boldface sequence agc, which was added to introduce an Eco47III site in the overlapping region in W4-F and W5-F, did not destroy the structure of the assembled sequence of the 11.88 kb DNA molecule in the final plasmid.

The overlapping primers for assembly of the 4.98 kb DNA molecule
B1-F: 5′-cgactctagaggatcGGACAAGATCTTGAAAGTGCTCGACGTCAATT-3′
B1-R: 5′-agctcggtacccgggGATCCATGAGAGGAC ^1^AACAAGGAAGATAGCTAT-3′
B2-F: 5′-GTCCTCTCATGGATC ^1′^TTGGTCCACAAGTG-3′
B2-R: 5′-agctcggtacccgggGATCCGCGGCCCGGC ^2^CTGGAACG-3′
B3-F: 5′-GCCGGGCCGCGGATC ^2′^TGCCTCG-3′
B3-R: 5′-agctcggtacccggg**gatcc**ACATCTCGCTCGGGTACCTGGTCGAAT-3′
The overlapping primers for assembly of the 7.09 kb DNA molecule
P1-F: 5′-ctctagaggatccccGGGTGGGGGAGGCAT ^3^CTGATATGCAGCAA-3′
P1-R: 5′-tcgagctcggtacccGCATGTGGACTGTGGAGGTGGCCAGTAATT-3′
P2-F: 5′-ctctagaggatccccGTCATGCATTCAGCCGTCAGAAAGGCTCAGA-3′
P2-R: 5′-ATGCCTCCCCCACCC ^3′^TACAACACCATGGCATCGATCGGATCAATCCTA-3′
The overlapping primers for assembly of the 11.88 kb DNA molecule
W1-F: 5′-ctctagaggatccccGGGAGCAAGAATTGT ^4^CGATGGAACCAGCT-3′
W1-R: 5′-tcgagctcggtacccCCTGTATAAGTTGAATTTGAATTTGCATGC-3′
W2-F: 5′-ctctagaggatccccGGGATAAATTAAACC ^5^ACGGTAATCTTCCAATAGAAC-3′
W2-R: 5′-ACAATTCTTGCTCCC ^4′^AACTTTGGGTATTTCAG-3′
W3-F: 5′-ctctagaggatccccGGGCGAGGCATCTTG ^6^GTAGCTGGCTT-3′
W3-R: 5′-GGTTTAATTTATCCC ^5′^AAGGTTACAGGTCTAGTAAT-3′
W4-F: 5′-ctctagaggatcccc**agc** GCTGACGACGATTAA ^7^TTGTGTGTGCAACG-3′
W4-R: 5′-CAAGATGCCTCGCCC ^6′^CTGCCCGAGA-3′
W5-F: 5′-ctctagaggatcccc**agc** GCTGCTTGCAAAACACTCTCCCTCTAAAAT-3′
W5-R: 5′-TTAATCGTCGTCAGC ^7′^CACGCTTTAATTTGTTC-3′

**Figure 3:**
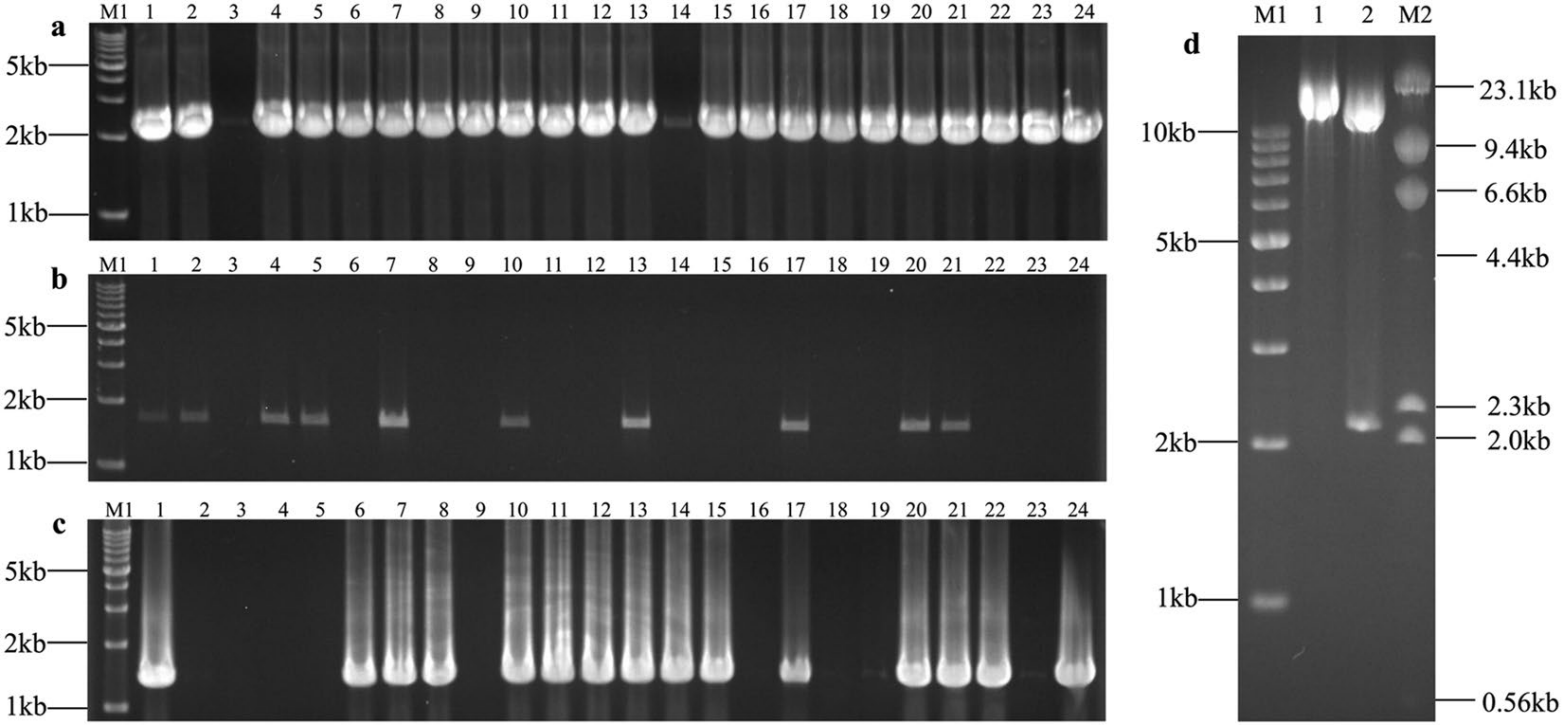
Identification of the assembly products. M1: TAKARA 1 kb DNA ladder; M2: TAKARA λ-HindIII digest. (**a**) Plasmids of pLDR-BF1(1)-(24) screened by PCR amplification directly from 24 colonies with primers B1-F and B1-R; the predicted band was 2025 bp. (**b**) Plasmids of pLDR-BF1~BF2(1)-(24) screened by PCR amplification directly from 24 colonies with the primers B2-F and B2-R; the predicted band was 1687 bp. (**c**) Plasmids of pLDR-BF1~BF3(1)-(24) screened by PCR amplification directly from 24 colonies with the primers B3-F and B3-R; the predicted band was 1362 bp. (**d**) 1: The final vector of pLDR-BF1~BF3 digested by BamHI; the predicted band was 14777 bp. 2: The final vector of pLDR-BF1~BF3 digested by SalI; the predicted bands were 12629 bp and 2148 bp.

We succeeded in seamlessly assembling the 4.98 kb DNA sequence into the pLDR vector using SSEA in 3 rounds of enzymatic reactions (Fig. 2b–d). After the first round, 22 colonies identified from 24 clones via PCR showed that the first fragment was successfully assembled into pLDR (Fig. 3a). Three colonies of pLDR-BF1(4), pLDR-BF1(8), and pLDR-BF1(9) were selected for sequencing; The sequence of BF1 in pLDR-BF1(4) was completely correct and the other 2 had mutations introduced by PCR. As expected, there was a BamHI site restored by splicing of the first stitching site and the overlapping region of pLDR in the successful assemblies (Supplementary Figure S3a). We digested pLDR-BF1(4) with BamHI to generate the assembly entrance and assemble the second fragment into pLDR-BF1(4). We identified 10 successful assemblies in a population of 24 colonies (Fig. 3b) and selected 4 colonies to sequence. The sequence of BF2 in 2 colonies of pLDR-BF1~BF2(1) and pLDR-BF1~BF2(4) were completely correct in 4 sequenced colonies. Remarkably, there was no nucleotide scar added at the junction between the first fragment of BF1 and the second fragment of BF2 (Supplementary Figure S3b), and there was a BamHI site restored at the junction of the second stitching site in the successful assemblies (Supplementary Figure S3c). The third fragment was then joined to pLDR-BF1~BF2(1) seamlessly in the third round of enzymatic reactions; 15 successful assemblies were obtained by PCR (Fig. 3c), and the 3 correct final plasmids pLDR-BF1~BF3(1), pLDR-BF1~BF3(6), and pLDR-BF1~BF3(10), were identified by sequencing from the 15 successful assemblies (Supplementary Figure S3d).

### Case 2: Assembly of a 7.09 kb DNA molecule by generating the blunt end of the assembly entrance

**Figure 4:**
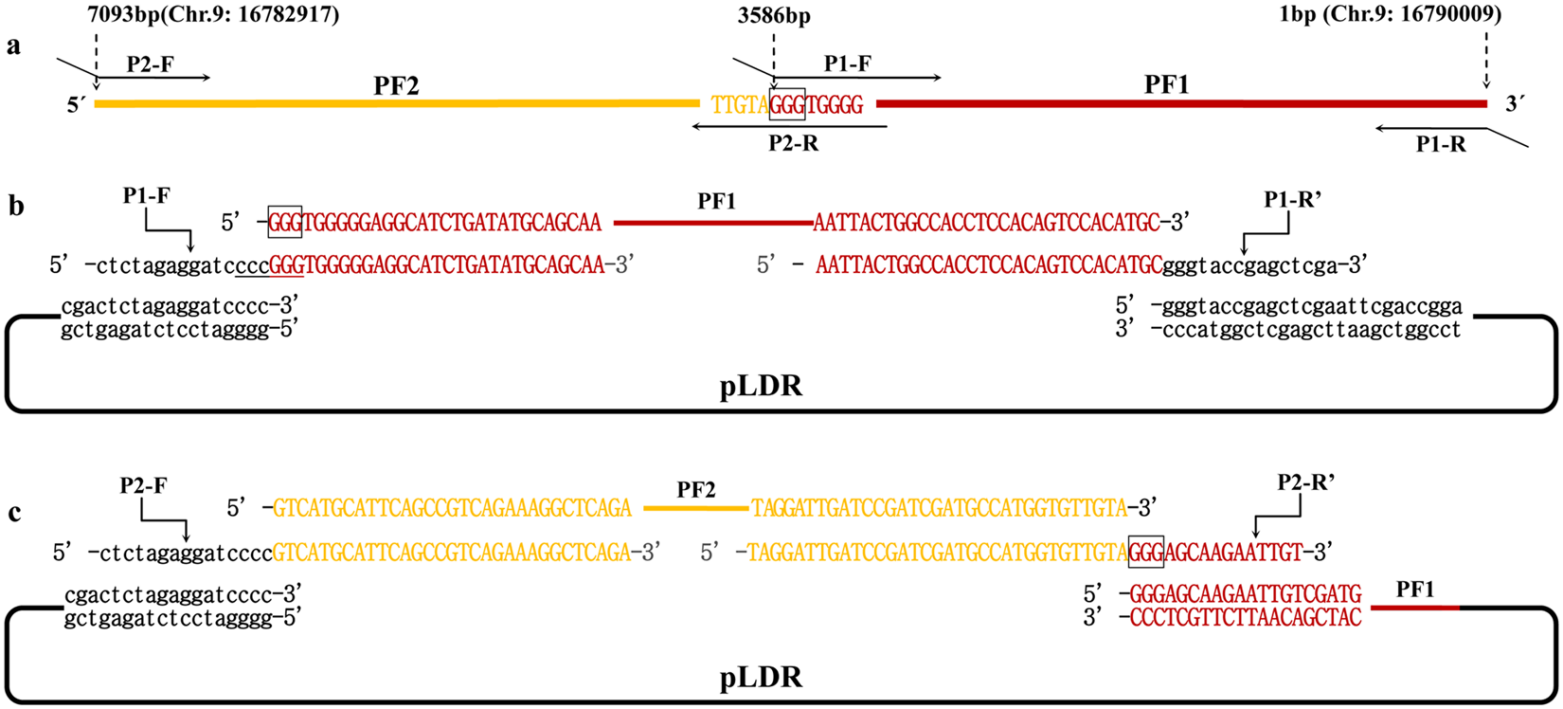
Assembly of a 7.09 kb DNA molecule by generating the blunt end of the assembly entrance. The stitching sites GGG are boxed. The black line and lowercase sequence represents pLDR. The red line and uppercase sequence represents the first fragment of PF1, and orange represents the second fragment of PF2. (**a**) The 7.09 kb DNA molecule was divided into two fragments by 1 stitching site GGG and the matching overlapping primers. (**b**) The first round of enzymatic assembly reactions for inserting PF1 is shown. The backbone vector of pLDR was digested by SmaI. The SmaI site was then eliminated in the forward primer of P1-R′ (P1-R′ was a reverse complement with P1-R), and a SmaI site (cccGGG) was restored by splicing the first stitching site and overlapping region in the reverse primer of P1-F. When the first fragment of PF1 obtained by PCR with primers of P1-F and P1-R was assembled into pLDR, there was only 1 SmaI site in the first round of the assembled products, which could be cut to generate the assembly entrance for the second round. (**c**) The second round of the enzymatic assembly reaction for inserting PF2 is shown. The intermediate plasmid of pLDR-PF1 was digested by SmaI. As for the first stitching site GGG in P2-R′ (P2-R′ was a reverse complement with P2-R), the second fragment of PF2 must join together with the first fragment of PF1 seamlessly.

**Figure 5:**
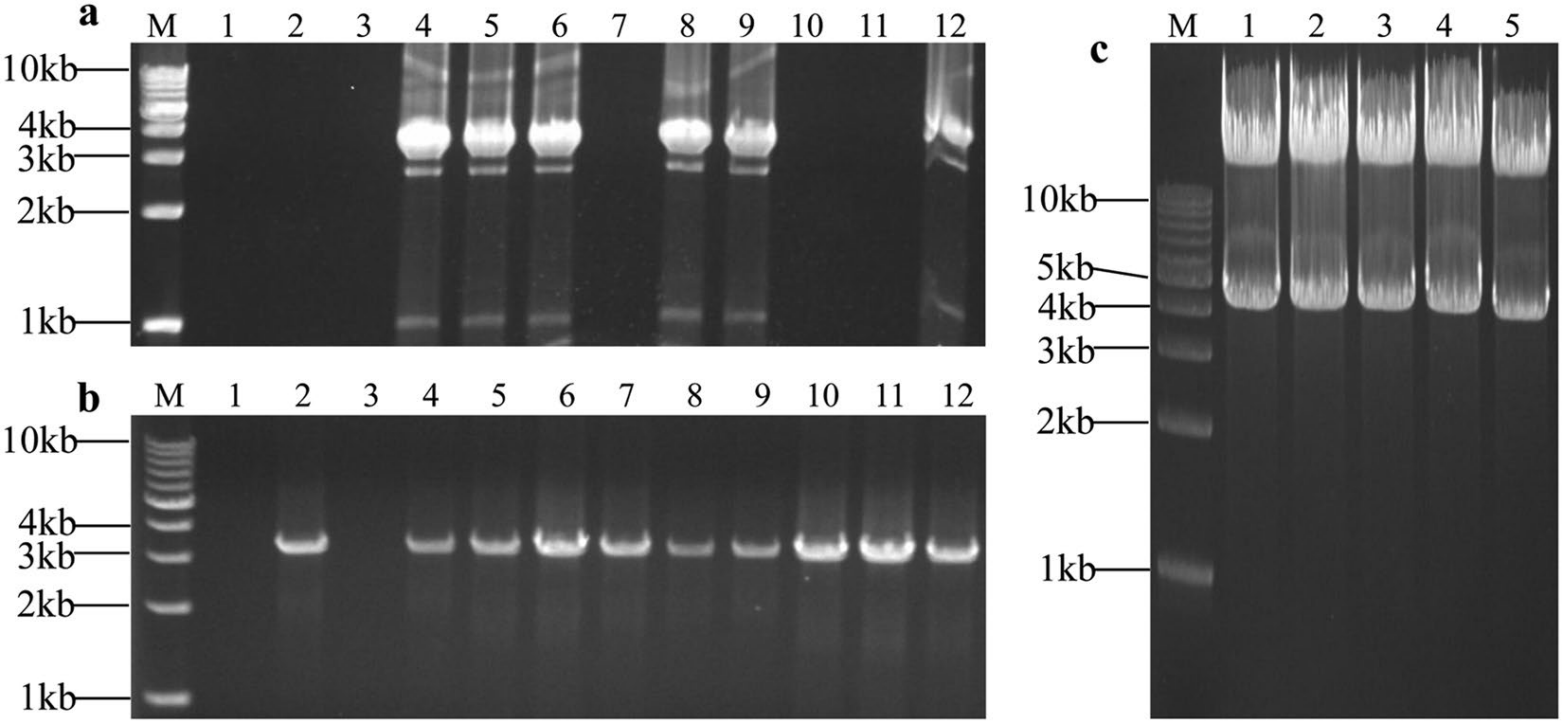
Identification of the assembly products. M: TAKARA 1 kb DNA ladder. (**a**) Plasmids of pLDR-PF1(1)–(12) screened by PCR amplification directly from 12 colonies with primers P1-F and P1-R; the predicted band was 3616 bp. (**b**) Plasmids of pLDR-PF1 ~ PF2(1)–(12) screened by PCR amplification directly from 12 colonies with the primers P2-F and P2-R; the predicted band was 3537 bp. (**c**) Digestion tests for the final vector of pLDR-PF1 ~ PF2. Five final plasmid DNAs analyzed by restriction digestion with KpnI; the predicted bands were 12615 bp and 4271 bp.

We showed the assembly of DNA in the 5′ → 3′ direction using BamHI to generate the sticky end of the assembly entrance in case 1. In case 2, we demonstrated the assembly of DNA in the 3′ → 5′ direction, employing restriction enzymes that generated the blunt end of the assembly entrance. A 7.09 kb genome DNA molecule from Chr.9: 16782917-16790009 in *Oryza sativa Japonica* L. was assembled into pLDR seamlessly in 2 rounds of enzymatic reactions (Fig. 4). When analyzing the restriction enzyme site, there were 7 blunt-end sites of SmaI, PmeI, SrfI, SnaBI, SspI, SwaI, and FspAI absent in the 7.09 kb genome DNA molecule (Supplementary Figure S4). SmaI, the common restriction enzymes and present at the multiple cloning sites of pLDR, was selected to linearize the plasmid to generate the assembly entrance. According to the 3′ → 5′ assembly direction, one stitching site GGG was selected to divide the 7.09 kb DNA molecule into 2 pieces of DNA fragments, with the size of the 2 fragments being 3507 bp and 3586 bp (Fig. 4a). Primers were designed using the same principles as for the first 3 fragments as described above (Table 2). After two rounds of enzymatic reactions, 10 candidate final plasmids were obtained according colony PCR (Fig. 5b). Five of them were digested by KpnI and all showed the predicted band sizes (Fig. 5c). The result of digestion shows that the 7.09 kb genome DNA molecule was successfully assembled into pLDR.

### Case 3: Assembly of an 11.88 kb DNA molecule by changing assembly entrance

**Figure 6:**
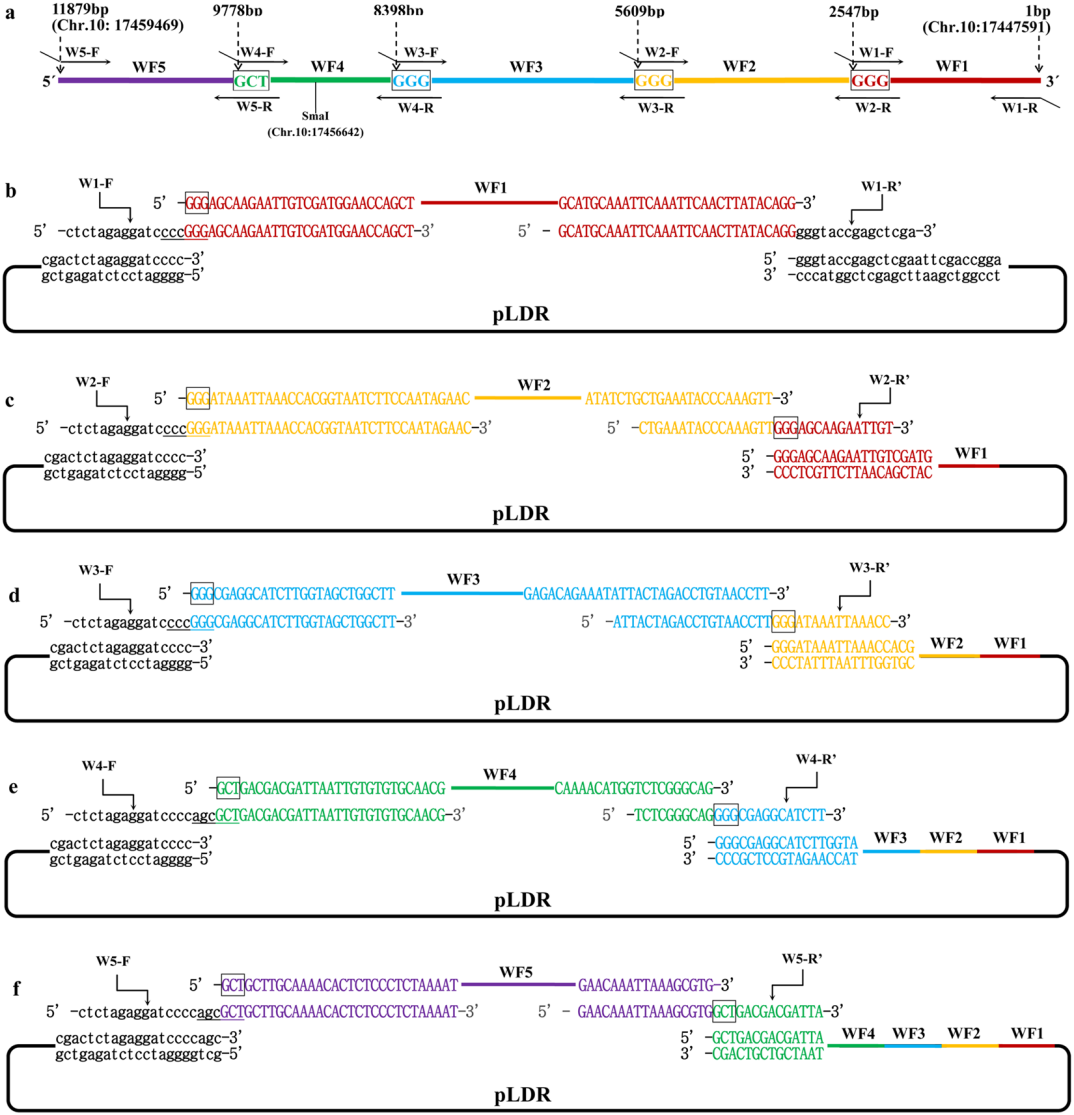
Assembly of the 11.88 kb DNA molecule by changing assembly entrance in the assembly process. The stitching sites GGG and GCT are boxed. The line and sequence shown in black represent pLDR, red represents the first fragment of WF1, orange represents the second fragment of WF2, blue represents the third fragment of WF3, green represents the fourth fragment of WF4, and purple represents the fifth fragment of WF5. The primer of W1-R′ was reverse complement with W1-R, as well as W2- R′ and W2-R, W3- R′ and W3-R, W4- R′ and W4-R, W5- R′ and W5-R. (**a**) The 11.88 kb DNA molecule was divided into five fragments by three stitching sites GGG and one stitching site GCT. (**b**) The first round of assembly reaction for inserting WF1. The pLDR was digested by SmaI. When WF1 was assembled into pLDR, there was only 1 SmaI site in the assembly products, which can be cut to generate the assembly entrance for the second round. (**c**) The second round of assembly reaction for inserting WF2. The pLDR-WF1 was digested by SmaI. As for the first stitching site GGG in W2-R′, WF2 must join together with WF1 seamlessly, and then there is only 1 SmaI site in the second round of assembled products. (**d**) The third round of assembly reaction for inserting WF3. The pLDR-WF1 ~ WF2 was digested by SmaI. As for the second stitching site GGG in W3-R′, WF3 must join together with WF2 seamlessly, and then there is only 1 SmaI site in the third round of assembled products. (**e**) The fourth round of assembly reaction for inserting WF4. The pLDR-WF1 ~ WF3 was digested by SmaI. An Eco47III was introduced by adding AGC additionally in the overlapping region of W4-F before the stitching site GCT. As for the third stitching site GGG in W4-R′, WF4 must join together with WF3 seamlessly, and then there is only 1 Eco47III site in the fourth round of assembled products. (**f**) The fifth round of assembly reaction for inserting WF5. The pLDR-WF1~WF4 was digested by Eco47III. The fifth fragment of WF5 seamlessly joins with WF4.

**Figure 7:**
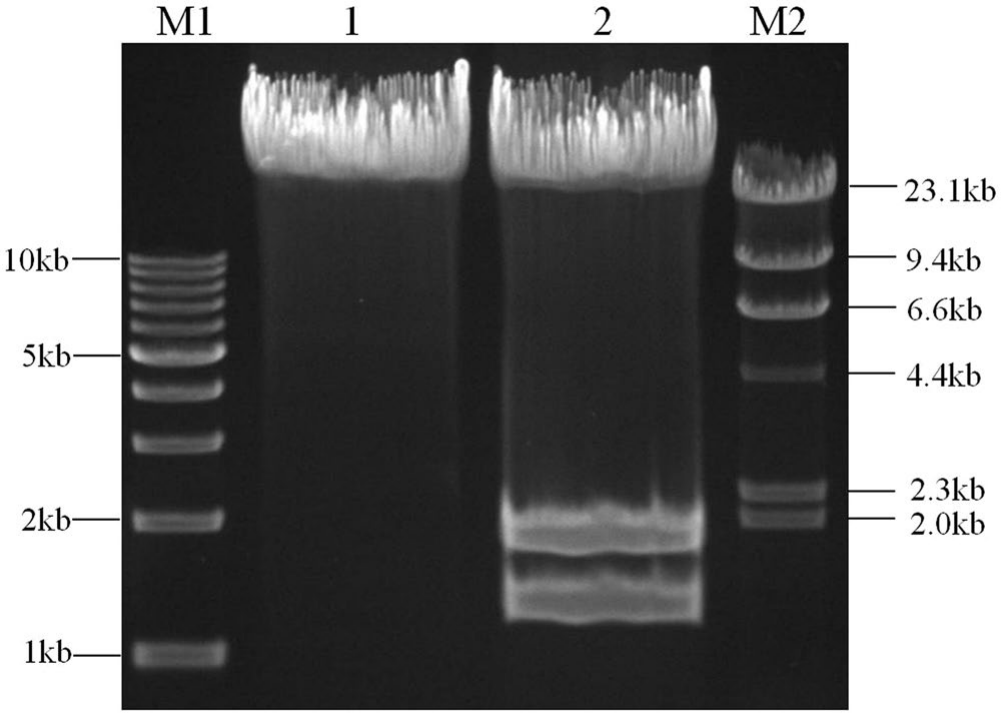
Identification of the final vector of pLDR-W1F1~WF5. M1: TAKARA 1 kb DNA ladder; M2: TAKARA λ-HindIII digest; 1: the plasmid of pLDR-WF1 ~ WF5 digested by Eco47III; the predicted band was 23505 bp; and 2: the plasmid of pLDR-WF1 ~ WF5 digested by SalI; the predicted bands were 20604 bp, 1715 bp and 1186 bp.

Since the required assembly entrance was not always present at the multiple cloning sites of the backbone vector, we demonstrated a solution in case 3 that changed the restriction sites used to generate an assembly entrance at the halfway point for assembly of an 11.88 kb genome DNA molecule (reverse complemented with Chr.10: 17447591-17459469 in *Oryza sativa Japonica* L.). When analyzing the restriction enzyme site, there were 24 restriction sites absent in the 11.88 kb DNA molecule (Supplementary Figure S5), but none of them were present at the multiple cloning sites of pLDR, so there was no suitable restriction site for use as an assembly entrance directly for assembly of the whole 11.88 kb DNA molecule. However there was one SmaI site present in the 11.88 kb genome DNA molecule located at Chr.10: 17456642-17456647 (Fig. 6a). Thus, we could first use SmaI to generate the assembly entrance for assembling the sequence after the SmaI site and then use Eco47III (selected from the 24 restriction sites described above) to assemble the sequence before the SmaI site. Because the 3′ → 5′assembly direction was determined according to the assembly order of the sequence, we selected 3 stitching sites GGG after the SmaI site and 1 stitching site GCT (Eco47III) before the SmaI site to segment the 11.88 kb DNA molecule into 5 fragments (Fig. 6a). Primers were designed using the same principles described above, then introduced into an Eco47III site in the overlapping region of W4-F to change the assembly entrance for the fifth fragment (Table 2). After 5 rounds of enzymatic reactions, the 11.88 kb DNA molecule was ligated into pLDR seamlessly (Fig. 6b–f). The digestion tests show that the 11.88 kb DNA molecule was successfully assembled into pLDR (Fig. 7).

## Discussion

Due to the limitations of the traditional digestion-ligation method, there are currently several assembly methods to construct large DNA molecule into plasmids for synthetic biology research^1^, including type IIS restriction enzymes-based assembly, site-specific recombination, and ligation-independent cloning methods, and each assembly strategy has its own advantages and disadvantages at different scales (Table 3). Type IIS restriction enzymes-based assembly methods overcome the drawbacks of introducing a scar sequence in traditional digestion-ligation, but the available type IIS restriction enzymes are rare and mostly 5nt or 6nt recognition sequences, with high frequency presented in the DNA sequence. Thus, it is still limited by the availability of unique restriction in some applications, similar to the traditional method^5–9^. Conversely, site-specific recombination does not rely on restriction sites but does leave a scar sequence in the final assembled plasmid^4,10,11^. The ligation-independent cloning method is a seamless strategy involving independent restriction sites; therefore, several versions have been developed. Among them, the polynary enzymatic assembly method is the most common and ideal for assembling large DNA molecules^12–19^. The type IIS restriction enzymes-based assembly does not leave scar sequence, site-specific recombination methods excel at overcoming the limitations of the restriction site and only the polynary enzymatic assembly method could adequately solve both the restriction site and scar sequence limitations. However, it appears that the larger the total size of the assembled inserts and the more fragments to be assembled, the lower the efficiency of the polynary enzymatic assembly method^22^. Additionally, it also suffers from the mutagenic artifact of PCR^20,23^, which disturbs the results of subsequent experimental. The limitations of the existing polynary enzymatic assembly method led us to design the Seamless Stack Enzymatic Assembly (SSEA) method, a binary enzymatic assembly mechanism based on a restriction-site-splicing strategy that enables the step-by-step joining of DNA fragments into a vector over multiple rounds of seamless enzymatic reactions.

**Table 3:** Advantages and disadvantages of the different DNA assembly methods.

	Seamless	Restriction site	Success rate	Influence of PCR on fidelity	Insert number in every step
Traditional digestion-ligation method	No	Rely on	Ok	Less	1
Type IIS restriction enzymes-based assembly	Yes	Rely on	Low	Less	Several
Site-specific recombination	No	Independent	Ok	Less	1 or several
Polynary enzymatic assembly	Yes	Independent	Low	More	Several
Seamless Stack Enzymatic Assembly	Yes	Low rely on	High	No	1

In our experience, there are 2 advantages of SSEA compared with polynary enzymatic assembly (Table 3). First, SSEA ligates only one fragment in each step and thus has a more controllable success rate than one-step cloning of multiple fragments. Second, with SSEA, a 100% accuracy rate is ensured because this method does not allow the assembly of a subsequent fragment until the previous one is correctly sequenced. Naturally, there is a minor limitation for generating the assembly entrance by common restriction enzymes when assembling a DNA molecule larger than 10-kb using SSEA. Nevertheless, we can select rare restriction sites or change restriction sites at the halfway point of SSEA to overcome this limitation (for example in case 3, the assembly entrance was changed from a SmaI site to an Eco47III site when assembling the fifth fragment). However, this limitation is minor compared to the advantages of high success rates and accuracy when using SSEA. The following are two proposals for using SSEA.

First, SSEA is given priority for common and blunt-end restriction enzymes. The common restriction enzymes are readily available and inexpensive; therefore, we selected common restriction enzymes as often as possible to linearize the plasmid. Also, we suggest utilizing restriction enzymes, which generate blunt ends, to linearize the plasmid. Compared to sticky-end restriction enzymes, it is easier to distinguish 2 stitching sites of the restriction site and design primers when using blunt end enzymes; for example, with the 6 nt recognition sequence of BamHI and SmaI, the 2 stitching sites of BamHI are part of GGATCC, with GGATC corresponding to the 5′ → 3′ assembly direction and GATCC corresponding to the 3′ → 5′ assembly direction, while the 2 stitching sites of SmaI are half of CCCGGG, with CCC corresponding to the 5′ → 3′ assembly direction and GGG corresponding to the 3′ → 5′ assembly direction. In addition, if the recognition sequences have the same number of nucleotides, the stitching sites of blunt-end enzymes appear at a higher frequency in the assembled sequence than the sticky-end enzymes because of the relative size difference. The more stitching sites present at the assembled sequence, the more options there are available for dividing the assembled sequence and the more convenient it is to design primers for the sequence. As in case 3, there are only 7 stitching sites GGATC and 3 stitching sites GATCC in the 11.88 kb DNA molecule, but there are 75 stitching sites GGG and 72 stitching sites CCC. Consequently, we selected SmaI as the assembly entrance for the first 4 rounds of the assembly of the DNA sequence.

Second, SSEA is suggested for selecting a suitable number of stitching sites to balance fragment size and enzymatic assembly steps. Since the locus and number of 2 stitching sites of restriction enzymes are different in the assembled sequence, we selected stitching sites that distribute relatively evenly so that the sequences are segmented into several fragments of almost equal size. For example, there were 6 stitching sites GGATC that distributed relatively evenly in the 4.98 kb sequence in case 1, while there were 5 stitching sites GATCC with comparatively concentrated distribution in the middle of the 4.98 kb sequence. This is why we selected stitching sites GGATC and assembly of the 4.98 kb sequence in the 5′ → 3′ direction. In addition, if there are more stitching sites, then the segmented fragments will be shorter, which are easier to amplify by PCR, but there will be more segmented fragments and an increase in the number of steps in the enzymatic assembly. Thus, we selected a suitable number of stitching sites to balance the size of the fragments and the number of steps for enzymatic assembly. In our experience, the optimal length of the segmented fragment is 1.5–3.5 kb.

In conclusion, SSEA is a flexible and efficient DNA enzymatic assembly strategy. We used this method to assemble 3 medium-sized DNA sequences and increased the success rate as much as possible. While the cost of SSEA is higher than that of polynary enzymatic assembly and SSEA involves more steps, the success rate and accuracy make the cost and time invested worthwhile in our experience. We anticipate that this method can be expanded upon in the future to accomplish assembly of larger DNA molecules and can become a common laboratory strategy.

## Methods

### Selection of appropriate restriction enzymes to generate the assembly entrance

We searched for all the restriction enzyme sites absent in the assembled DNA sequence with the DNAssist 2.0 program and selected a common or blunt enzyme to linearize the plasmid. The selected restriction enzymes were used to linearize the plasmid, providing an entrance for assembly of the DNA fragment. The linearized plasmids included portions of the restriction site nucleotides on either end. For example, the plasmid of pLDR cut by BamHI retained GGATC or GATCC (lined up with the ends) on either end in the 5′-3′ sense strand sequence. The nucleotides of GGATC and GATCC were termed the 2 stitching sites of BamHI. We determined the DNA assembly direction based on the selected stitching site. For example, if we wanted to utilize BamHI to generate the assembly entrance and to use GGATC as the stitching site in the assembled DNA sequence, we assembled DNA in the 5′ → 3′ direction, and if we selected GATCC as the stitching site, the assembly direction was 3′ → 5′ (Table 1).

### Design of overlapping primers according to the locus of stitching sites and assembly direction

We selected several stitching sites as the boundaries to divide the large DNA molecule into several shorter assembly fragments (Figs 2A, 4A and 6A). We then designed a set of overlapping primers (Table 2) to amplify assembly fragments using the following principles of primer design: (i) All primers include a priming fragment-specific sequence and overlapping sequences of adjacent assembly fragments. (ii) To achieve efficient amplification of assembly fragments, we suggest the priming fragment-specific sequence of 25–35 nt with a Tm equal to or greater than 68 °C, with an overlapping sequence of 15–25 nt. (iii) We could edit the overlapping sequence to eliminate or restore the pre-existing restriction site or to introduce a new restriction site in primers to modify their amplicon, which does not destroy the original sequence when assembled into the backbone vector. For example, the primer of B1-F is the eliminated BamHI site and the primer of B1-R restores the BamHI site by splicing the first stitching site GGATC and adjacent nucleotide of C in the overlapping region (Table 2). The primer of W4-F was introduced at an Eco47III site by splicing AGC and the stitching site GCT, with the AGC nucleotides added in the overlapping region (Table 2).

### PCR amplification, enzymatic reaction, and transformation

The assembly fragments were amplified from *Oryza Sativa Japonica* L. DNA templates using the primers described above and KOD FX DNA Polymerase. The PCR reactions were prepared according to the manufacturer′s recommendations using the following cycles: 3 min at 98 °C followed by 3 cycles with 20 s at 98 °C, 1–3 min at 65 °C and 35 cycles with 15 s at 98 °C, 1–3 min at 68 °C, and a final extension step of 5 min at 68 °C. 0.5–1 μg of plasmid DNA was digested by the assembly entrance enzyme in 50 μl reaction solution containing 5 μl 10 × FastDigest buffer and 5 μl of FastDigest™ enzyme (ThermoFisher), with 30 min of incubation time. The linearized plasmid and PCR products were subjected to gel electrophoresis and purified using the OMEGA gel extraction kit. All of the enzymatic reactions were performed using 0.1 pmol of DNA for each part and were set up on ice. In-Fusion and Gibson assembly reactions were performed as described^16,18^, with 45 min of incubation time. We transformed 5 μl of the assembly product in chemically competent *E. coli* cells. Transformation reactions were run following the Tans1-T1 Phage Resistant Chemically Competent Cell Transformation Protocol. Desired plasmids were verified by colony PCR or by gel electrophoresis following analytical restriction digestion and sequencing.

## Electronic supplementary material

Supplementary information
