# Supplementary material for: In Vitro Seamless Stack Enzymatic Assembly of DNA Molecules Based on a Strategy Involving Splicing of Restriction Sites
Source: Sci Rep. 2017 Oct 27;7:14261. doi: 10.1038/s41598-017-14496-5 (PMC5660187; doi:10.1038/s41598-017-14496-5)
Supplement: Supplementary file 1 — Supplementary information [file 41598_2017_14496_MOESM1_ESM.pdf]

# *In Vitro* Seamless Stack Enzymatic Assembly of DNA Molecules Based on a Strategy Involving Splicing of Restriction Sites

Dong Yu[1,2,3], Yanning Tan[1,2], Zhizhong Sun[1,2,3], Xuewu Sun[1,2], Xiabing Sheng[1,2], Tianshun Zhou[1,4], Ling Liu[1,4], Yi Mo[1,3], Beibei Jiang[1,4], Ning Ouyang[1,4], Xiaolin Yin[1,4], Meijuan Duan[*3], and Dingyang Yuan[*1,2,3,4]

[1]State Key Laboratory of Hybrid Rice, Hunan Hybrid Rice Research Center, 736 Yuanda Rd, Changsha, 410125, PRC, [2]Hunan Academy of Agricultural Sciences, 892 Yuanda Rd, Changsha, 410125, PRC, [3]College of Bioscience and Biotechnology, Hunan Agricultural University, 1 Nongda Rd, Changsha, 410128, PRC, [4]Long Ping Branch, Graduate School of Hunan University, 892 Yuanda Rd, Changsha, 410125, PRC

*Correspondence and requests for materials should be addressed to D. Y. (yuandingyang@hhrrc.ac.cn) or M.D. (duanmeijuan@163.com)

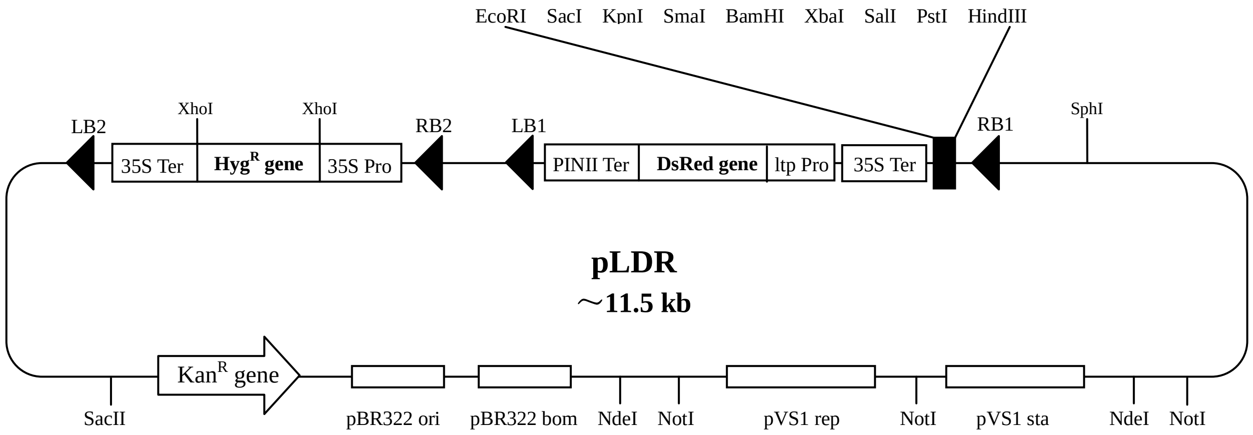


**Supplementary Figure S1.** The vector of pLDR and its multiple cloning site.

```
Absent sites

AarI  CACCTGC,4,8              NheI  G/CTAGC
AceII  GCTAG/C                NotI  GC/GGCCGC
AclI  AA/CGTT                 NruI  TCG/CGA
AflII  C/TTAAG                PacI  TTAAT/TAA
AgeI  A/CCGGT                 PciI  A/CATGT
AloI  GAACNNNNNNTC/C          PflMI  CCANNNN/NTGG
AlwNI  CAGNNN/CTG             PmeI  GTTT/AAAC
AscI  GG/CGCGCC               PmlI  CAC/GTG
AseI  AT/TAAT                 PsiI  TTA/TAA
AsiSI  GCGAT/CGC              PsrI  GAACNNNNNNTA/C
AvrII  C/CTAGG                PstI  CTGCA/G
BaeI  ACNNNNGTAYC,12,7        PvuII  CAG/CTG
BamHI  G/GATCC                SanDI  GG/GWCCC
BloHII  CTGCA/G               SbfI  CCTGCA/GG
BsaI  G/GTCTC                 SfiI  GGCCNNNN/NGGCC
BstZ17I  GTA/TAC              SnaBI  TAC/GTA
BtsI  GC/AGTG                 SpeI  A/CTAGT
Eco47III  AGC/GCT             SrfI  GCCC/GGGC
FseI  GGCCGG/CC               SspI  AAT/ATT
FspAI  RTGC/GCAY              StuI  AGG/CCT
FspI  TGC/GCA                 SwaI  ATTT/AAAT
HpaI  GTT/AAC                 XbaI  T/CTAGA
```

**Supplementary Figure S2.** The absent restriction sites in the 4.98 kb DNA sequence.

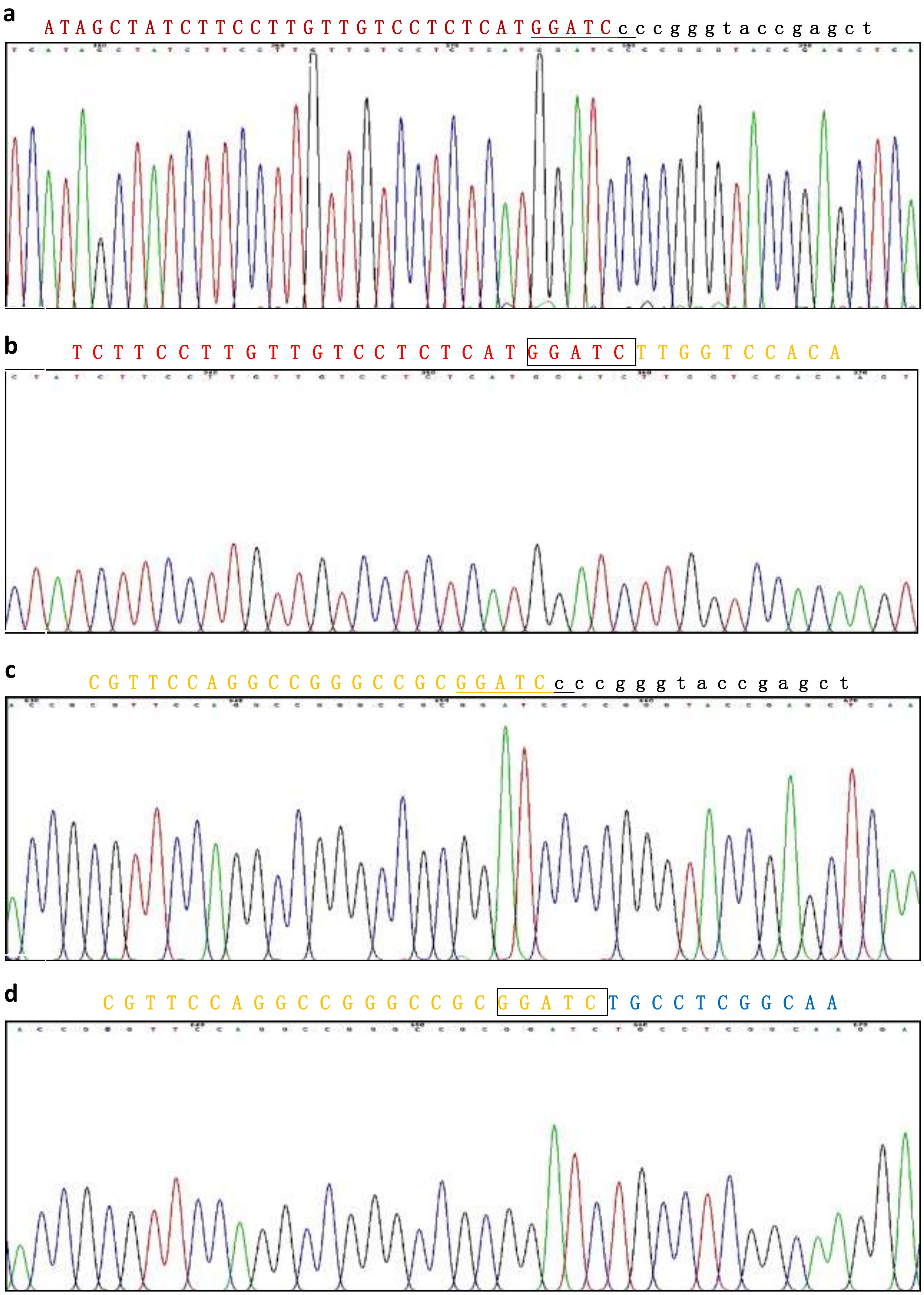


**Supplementary Figure S3.** The sequencing results of the junction sites in case 1. The stitching sites GGATC are boxed. The lowercase letters shown in black represents sequence of pLDR. The uppercase letters shown in red

represents sequence of the first fragment of BF1, orange represents sequence of the second fragment of BF2, and blue represents sequence of the third fragment of BF3. (a) There was a BamHI site (GGATCc) restored by splicing of the first stitching site GGATC and adjacent nucleotide of c at the end of linearized pLDR in the first successful assemblies. (b) There was no nucleotide scar added at the junction between the first fragment of BF1 and the second fragment of BF2 in the second successful assemblies. (c) There was a BamHI site (GGATCc) restored by splicing of the second stitching site GGATC and adjacent nucleotide of c at the end of linearized pLDR in the second successful assemblies. (d) There was no nucleotide scar added at the junction between the second fragment of BF2 and the third fragment of BF3 in the third successful assemblies.

```
Absent sites

AclI   AA/CGTT                RsrII   CG/GWCCG
AgeI   A/CCGGT                SanDI   GG/GWCCC
AscI   GG/CGCGCC              SbfI   CCTGCA/GG
AsiSI   GCGAT/CGC             SfiI   GGCCNNNN/NGGCC
AvrII   C/CTAGG               SmaI   CCC/GGG
BaeI   ACNNNNGTAYC,12,7       SnaBI   TAC/GTA
BspEI   T/CCGGA               SrfI   GCCC/GGGC
BstBI   TT/CGAA               Sse8647I   AG/GWCCT
FspAI   RTGC/GCAY             SspI   AAT/ATT
NotI   GC/GGCCGC              SwaI   ATTT/AAAT
PacI   TTAAT/TAA              TaqII   GACCGA,11,9
PmeI   GTTT/AAAC              XmaI   C/CCGGG
PsrI   GAACNNNNNNTA/C         XmnI   GAANN/NNTTC
```

**Supplementary Figure S4.** The absent restriction sites in the 7.09 kb DNA sequence.

```
Absent sites

AatII   GACGT/C               MfeI   C/AATTG
ApaI   GGGCC/C                NotI   GC/GGCCGC
AscI   GG/CGCGCC              PmeI   GTTT/AAAC
AsiSI   GCGAT/CGC             PspOMI   G/GGCCC
BaeI   ACNNNNGTAYC,12,7       RsrII   CG/GWCCG
BssHII   G/CGCGC              SanDI   GG/GWCCC
BstXI   CCANNNNN/NTGG         SbfI   CCTGCA/GG
BtrI   CACGTC,-3,-3           SfiI   GGCCNNNN/NGGCC
EagI   C/GGCCG                SpeI   A/CTAGT
Eco47III   AGC/GCT            SrfI   GCCC/GGGC
FseI   GGCCGG/CC              Sse232I   CG/CCGGCG
FspAI   RTGC/GCAY             ZraI   GAC/GTC
```

**Supplementary Figure S5.** The absent restriction sites in the 11.88 kb DNA sequence.
